# Precore Mutation of Hepatitis B Virus May Contribute to Hepatocellular Carcinoma Risk: Evidence from an Updated Meta-Analysis

**DOI:** 10.1371/journal.pone.0038394

**Published:** 2012-06-01

**Authors:** Yun Liao, Xin Hu, Jie Chen, Bei Cai, Jiangtao Tang, Binwu Ying, Haiqing Wang, Lanlan Wang

**Affiliations:** 1 Department of Laboratory Medicine, West China Hospital, Sichuan University, Wuhou District, Chengdu, China; 2 Department of Neurosurgery, West China Hospital, Sichuan University, Wuhou District, Chengdu, China; 3 Department of Liver and Vascular Surgery, Liver Transplantation Center, West China Hospital of Sichuan University, Chengdu, Sichuan Province, China; University of Modena & Reggio Emilia, Italy

## Abstract

**Background:**

Studies focused on the correlation of mutations in the genome of Hepatitis B Virus (HBV) like Pre-S mutation, Basal Core promoter (BCP), Enhancer II (EnhII), especially Precore mutation, with the risk of hepatocellular carcinoma (HCC) have triggered stiff controversies. With an increasing number of studies in this field recently, we conducted this meta-analysis to appraise the correlations.

**Methods:**

We searched the commonly used databases both in English and Chinese till February 1^st^, 2012. Meta-analysis was performed in fixed/random-effects models using STATA 10.0. Publication bias was examined through Egger's test and Begg's funnel plot.

**Results:**

In total, 85 case-control studies were included involving 16745 HBV-infected patients, of whom 5781 had HCC. Statistically significant correlations were observed in Precore mutation G1896A (OR = 1.46, 95% confidence interval [CI] = 1.15–1.85, P_OR_ = 0.002), G1899A (OR = 3.13, 95%CI = 2.38–4.13, P_OR_<0.001) and Pre-S mutation especially Pre-S1 deletion (OR = 2.94, 95%CI = 2.22 to 3.89) and Pre-S2 deletion (OR = 3.02, 95%CI = 2.03 to 4.50). Similar correlation existed between BCP double mutation A1762T/G1764A, T1753V, C1653T and HCC. In subgroup analysis, the Asians, genotype C or HBeAg positive patients with certain above mutations may be more susceptible to HCC. Besides, the mutations like G1896A and BCP double mutation may be associated with the progression of the liver diseases.

**Conclusions:**

Precore mutation G1896A, G1899A, deletions in Pre-S region as well as the other commonly seen mutations correlated with the increased risk of HCC, especially in Asians and may predict the progression of the liver disease.

## Introduction

Hepatocellular carcinoma (HCC) is the primary histological subtype of the liver cancer which ranks the fifth most prevalent cancer worldwide and the second leading cause of the cancer mortality [1]. The main factors attributing to HCC encompass the chronic hepatitis B virus (HBV) and/or hepatitis C virus (HCV) infection etc., while the main reason for HCC comes to the chronic infection with HBV [2], [3].

HBV contains an incomplete double-stranded DNA genome consisting of four main regions: the PreS/S region (nucleotides or nt 2854–155), the enhancer II(EnhII; nt 1636–1744) region, basal core promoter (BCP, nucleotides 1751–1769) region and the precore region. It has been demonstrated that mutations in the HBV genome which is pertinent to HCC invariably occur at the PreS region, EnhII, BCP and precore region. Those mutations include G1613A, C1653T in the EnhII region; T1753V, the double mutation A1762T/G1764A at the BCP region, G1896A and G1899A in the precore region [4], [5]. Several prospective studies demonstrated that patients who had A1762T/G1764A double mutation were more predisposed to HCC than those with the wild type [4], [6]. Similar associations with HCC can be found in mutations in the precore region as well as that in the EnhII region [7], [8]. The PreS mutation generally presents in form of deletions [9]–[11]. And it primarily comprises of PreS1 deletion and PreS2 deletion. The PreS1 and PreS2 regions are vital for the interaction with the host immune responses [12]. Thus the mutations occur in the PreS region would contribute to the inefficient immune response and ultimately lead to hepatocarcinogenesis. Therefore, ascertaining the relationship between the mutations in HBV genome and the occurrence of HCC may prompt the advancement in predicting the occurrence of HCC, screening the genetic predisposition as well as developing the clinical treatment regimes.

However, several published studies showed a negative association between these mutations and the onset of HCC [13]–[15]. To illuminate the controversy, a related meta-analysis was performed and demonstrated a positive relationship between the mutations like BCP double mutation, Pre-S mutation, C1653T and T1753V and the occurrence of HCC, while no significance was observed in Precore mutation G1896A [16]. Till now, much more studies (around 40 more case-control studies) focusing on the correlation of HBV mutations with HCC have emerged and increasing studies demonstrated a significant correlation between Precore G1896A and the risk of HCC [4], [17]–[19]. Besides, a new mutation pattern G1899A in precore region appears to significantly correlate with the incremental risk of HCC [4], [17], [20], [21] as well. Moreover, for Pre-S mutation, more studies focused on Pre-S1 deletion and Pre-S2 deletion [9], [22], [23] respectively and it would be of great help in discerning the contribution each mutation in Pre-S region made to HCC risk through analyzing their effects respectively. Thus it's imperative to update this meta-analysis to give us a more comprehensive understanding on those mutations with HCC risk. Therefore we reappraised the relationship between the ubiquitous mutations and the risk of suffering from HCC in this study, with great attention paying to Precore G1896A and the HCC risk.

## Materials and Methods

### Search strategy for the original article

A comprehensive search was conducted till February 1^st^ 2012 using the following databases: PUBMED, Embase, Chinese Biomedical Literature Database (CBM) and China National Knowledge Infrastructure (CNKI). Medical Subject Heading (MeSH) terms were of the priority in setting the strategy. The key words were “hepatitis b virus” “mutation” and “hepatocellular carcinoma”. In addition, we scrutinized the reference citations in the retrieved articles so as not to miss any additional eligible studies.

### Criteria for article screening

Studies were included if they met the following criteria: 1) the article assessed the association between HBV mutations and HCC; 2) study design was a case-control study; 3) the diagnosis of each stage of liver disease conformed to the American Association for the Study of Liver Disease [12], [24]; 4) odds ratio with the 95% confidence interval was reported or could be figured out through the available data. The unpublished reports like the conference abstracts were not included. We excluded articles that had no control group, case and control number less than 10 respectively, as well as patients who have co-infection with HCV and/or HIV or patients with liver disease caused by other non-infection reasons. As for the studies conducted by the same author, if the two inclusion time overlapped for more than 30% of the study time or all the patients were from the same region, we only adopted the most recent article or the one with the larger case number. While those studies written by the same author focused on distinct mutations were still included.

### Data collection

The data was collected by two members (Yun Liao and Xin Hu) independently, using the standardized form. When any discrepancy occurred, we consulted with the other investigators until the consensus was reached. The following information was extracted: first author's name, publication time, country, ethnicity, study design, sequencing method, number of cases (patients who have developed HCC) and controls (patients without HCC), mean age, male proportion, genotype distribution and mutation sites. Still, we extracted data based on subgroups like ethnicity, genotypes and HBeAg statuses etc.

### Statistical analysis

The impact the mutations have on HCC were estimated by summary odds ratios (OR) and their corresponding 95%CI. The overall effect was appraised through the Z test which could be deemed significant if the P value was less than 0.05. The heterogeneity for the included articles was evaluated using Cochran's Q test, I^2^ statistics (the heterogeneity could be accepted if P>0.1 and I^2^≤50%) and Galbraith plot for heterogeneity. If the value of I^2^ statistics was less than 50% or the P value is more than 0.1, the fixed-effects model can be tapped, otherwise, random-effects model be used. Galbraith plot was used to determine the main sources of the heterogeneity. Begg's funnel plot and Egger's test were performed to examine the publication bias. All the statistical analyses were performed using STATA (version 10.0). All tests were two sided.

## Results

### Study selection

**Figure 1:**
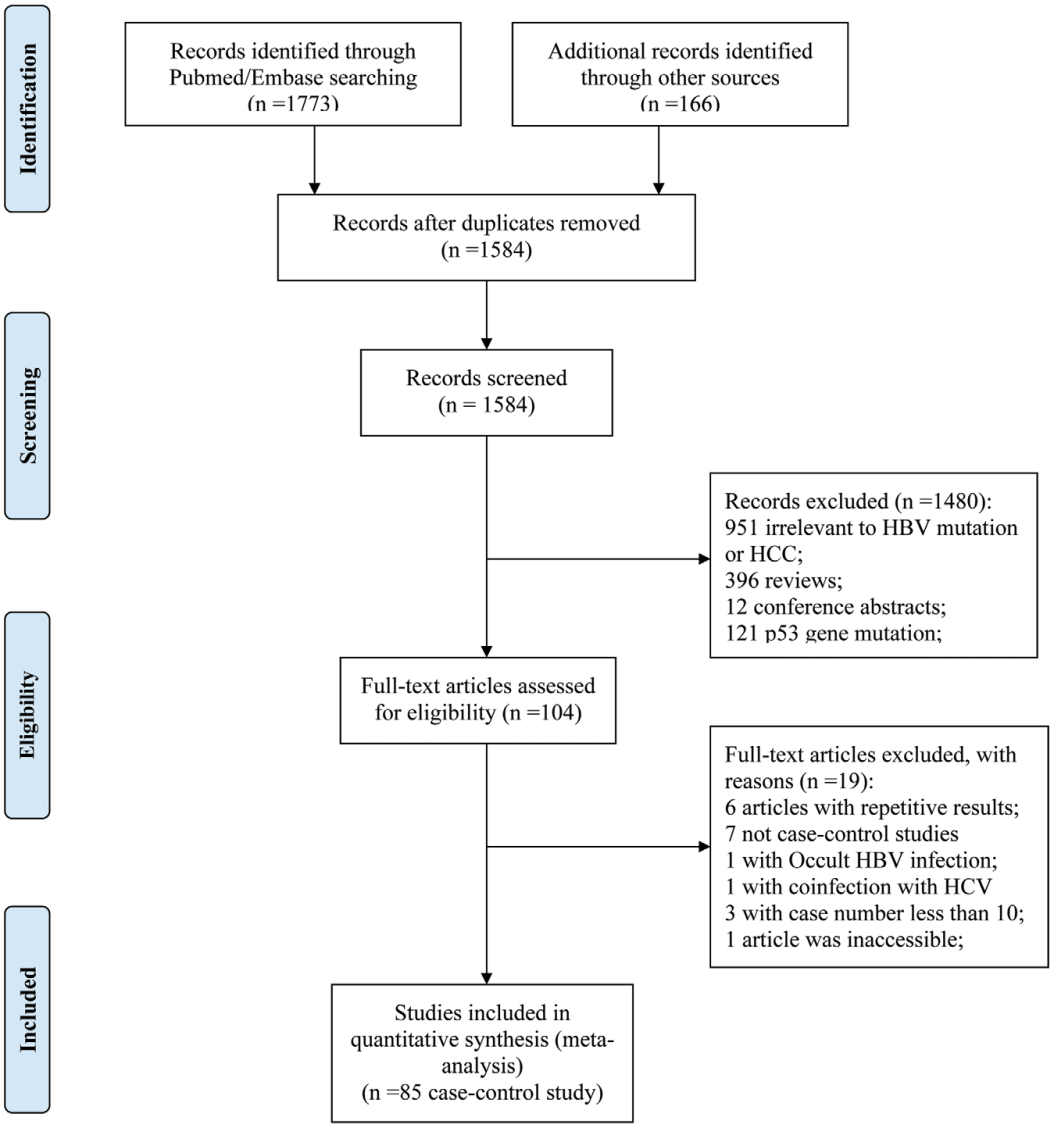
Flow chart for article screening in the meta-analysis. After a comprehensive screening, a total of 85 case-control studies were identified.

The flow diagram (See Figure 1) describing the screening process was modified according to the PRISMA Statement [25]. After skimming the titles and abstracts, 104 articles were included for full text view. Among them, seven articles were not case-control studies [26]–[32], one with co-infection patients [33], one with occult HBV infection [34], three with case and control number less than 10, respectively [35]–[37], one article was inaccessible [38] and six other studies with repetitive results (reference not shown). Finally, 85 case-control studies were included, among which, 43 case-control studies focused on G1896A, 56 studies on A1762T/G1764A, 24 studies on Pre-S mutation, 27 studies on T1753V and 23 studies on C1653T. The characteristics of the qualified articles were summarized in Table S1.

### Precore mutations and HCC risk

**Figure 2:**
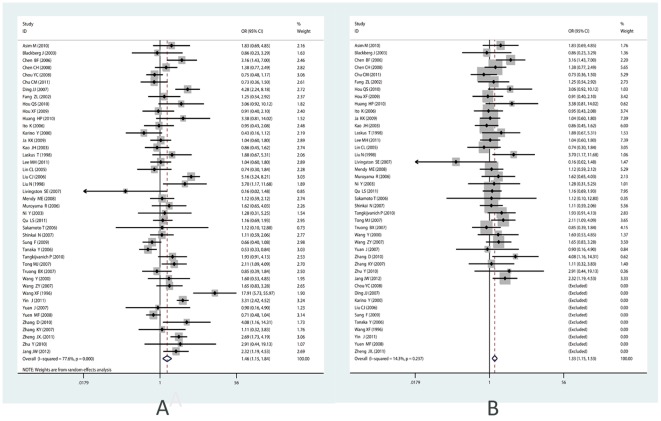
Forest plot for the correlation between Precore mutation G1896A and HCC risk. (A) Pooled odds ratio for correlation of Precore G1896A with HCC (before adjustment for heterogeneity). (B) Pooled odds ratio for association between G1896A and HCC risk after adjustment for heterogeneity. (For the forest plot, the black diamond represented the OR estimate for each study and the size of the grey area reflected the weight in the pooled analysis; the horizontal line indicates the 95% confidence interval (CI); the white diamond represented the pooled odds ratio).

**Figure 3:**
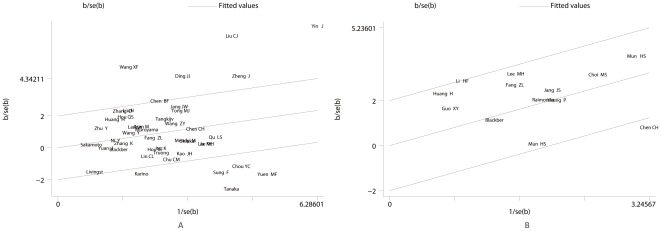
Galbraith plots for heterogeneity test of G1896A and Pre-S2 deletion. (A) Galbraith plot of the association between precore G1896A and HCC risk (The studies outside the range between −2 and 2 were seen as the outliers and the major source of heterogeneity); (B) Galbraith plot of the correlation between Pre-S2 deletion and HCC risk.

For the controversial Precore mutation G1896A, 43 studies were included in the meta-analysis. Heterogeneity existed among all the studies (I^2^ = 77.6%, P<0.001), thus the random-effects model was used. Significant correlation was found between G1896A and the occurrence of HCC (summary OR = 1.46; 95%CI = 1.15–1.84) (See Figure 2A). When performing Galbraith Plot for Heterogeneity, we observed ten outliers (See Figure 3A) as the main source of heterogeneity. Then we omitted these ten studies and reappraised the correlation. Still significant correlation was found with no statistically heterogeneity existed (OR_adjusted_ = 1.33, 95%CI = 1.16–1.53, I^2^ = 14.3%, P_heterogeneity_ = 0.237) (See Table 1; Figure 2B). In the subgroup analysis by ethnicity, the results indicated that G1896A correlated with an increased risk among the Asians (OR = 1.47, 95% CI = 1.16–1.92,) and even after adjustment for heterogeneity (OR = 1.33, 95% CI = 1.14–1.54), while no such correlation was found in Caucasians and Africans (See Table 1). For subgroup analysis by genotype, especially in genotype C, great heterogeneity existed (I^2^ = 85.4%, P = 0.107). According to Galbraith plot, studies conducted by Lyu H [39], Yin J [4], and Tanaka [40] were three outliers (data not shown). After omitting these three, significant correlation was observed (OR = 1.34, P_OR_ = 0.041, I^2^ = 39.2%). While for HBeAg status subgroup, we didn't observe any significant association, but the trend toward significant association became increasingly obvious (See Table 1).

**Table 1:** Summary of the odds ratio and its 95% Confidence Interval in the meta-analysis.

Mutations/Subgroup analysis	No. of study	OR (95%CI)	P_OR_ *	I^2^	PH#	Effect model	T§	P_egger_ §
**Precore G1896A**								
** Total**	43	**1.458 (1.153, 1.845)**	**0.002**	77.6%	0.000	R 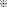	−0.14	0.891
** Total (adjustment** ♂ **)**	33	**1.328 (1.155, 1.528)**	**0.000**	14.3%	0.237	F	1.01	0.319
*Subgroup analysis by ethnicity*								
** Asians**	38	**1.492 (1.158, 1.923)**	**0.002**	79.5%	0.000	R		
** Asians(adjustment)**	28	**1.325 (1.140, 1.540)**	**0.000**	12.5%	0.278	F		
Caucasian	1	0.162 (0.018, 1.479)	0.107	/	/	-		
African	2	1.291(0.751, 2.219)	0.355	0.0%	0.401	F 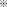		
Mixed	2	1.774 (0.988, 3.187)	0.055	27.6%	0.240	F		
*Subgroup analysis by genotype*								
Genotype B	4	2.665 (0.874, 8.127)	0.085	79.9%	0.002	R		
Genotype C	11	1.540(0.911, 2.603)	0.107	85.4%	0.000	R		
** Genotype C(adjustment)**	8	**1.344 (1.013,1.784)**	**0.041**	39.2%	0.118	F		
Genotype D	2	0.750 (0.038,14.972)	0.851	/	/	F		
*Subgroup analysis by HBeAg*								
HBeAg negative	4	1.339 (0.925, 1.940)	0.122	26.5%	0.253	F		
HBeAg positive	4	1.382 (0.804, 2.376)	0.242	43.9%	0.148	R		
**Precore G1899A**								
** Total**	**11**	**3.134 (2.376, 4.134)**	**0.000**	33.0%	0.135	F	0.63	0.543
*Subgroup analysis by ethnicity*								
** Asian**	**10**	**3.221 (2.423, 4.282)**	**0.000**	37.3%	0.110	F		
African	1	1.895 (0.565, 6356)	0.301	/	/	F		
**Pre-S mutaton**								
** Pre-S1 deletion**								
** Total**	**11**	**2.939 (2.222, 3.886)**	**0.000**	3.9%	0.411	F	−1.66	0.132
** Pre-S2 deletion**								
** Total**	**13**	**3.017 (2.025, 4.496)**	**0.000**	43.3%	0.048	R	**2.43**	**0.033**
** Total(adjustment)**	**12**	**3.510 (2.605, 4.729)**	**0.000**	16.8%	0.279	F	1.74	0.113

* P_OR_ value for the odds ratio.

# P_H_ the P value of the Heterogeneity test.

§ T for Egger's test, P_egger_, the P value for Egger's test.

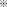
R for the Random-effects model, F for the Fixed-effects model.

♂ Adjustment for Heterogeneity by omitting the most obvious outliers.

**Figure 4:**
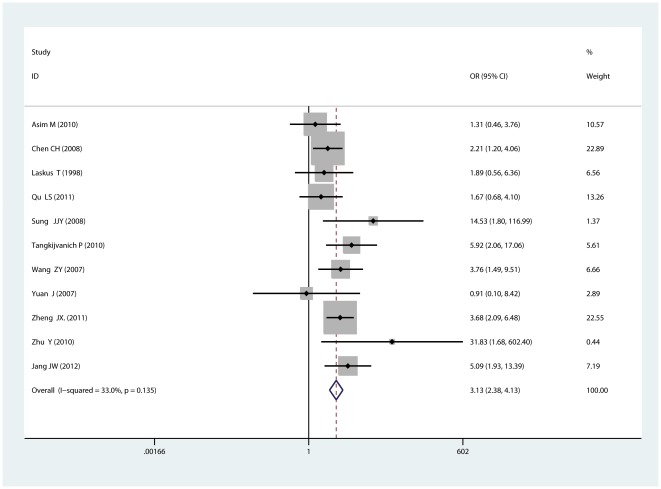
Forest plot for the correlation between Precore mutation G1899A and HCC risk.

For another new mutation G1899A in Precore region, 11 studies including 693 cases and 953 controls were included. No statistically significant heterogeneity existed (I^2^ = 33.0%, P_heterogeneity_ = 0.135) and the fixed-effects model was used. Results showed that G1899A correlated with an increased risk of HCC (OR = 3.13, 95%CI = 2.38–4.13) (See Figure 4). In subgroup analysis by ethnicity, a significant association was found to exist between G1899A and HCC in Asians (OR = 3.22, 95%CI = 2.42–4.28). While only one study [41] focused on the Africans and no correlation was found.

### Pre-S deletions and HCC risk

**Figure 5:**
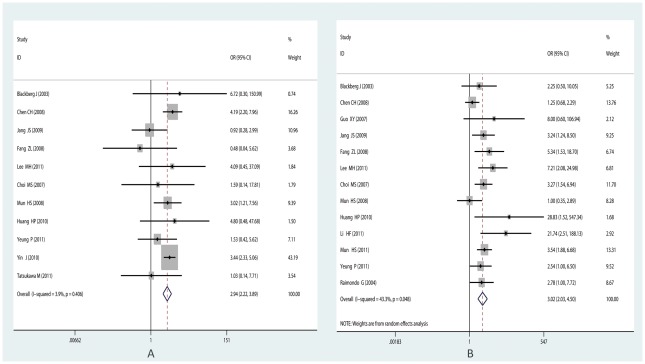
Forest plot for the correlation between Pre-S deletions and HCC risk. (A) Pooled odds ratio for correlation of Pre-S1 deletion and HCC risk using fixed-effects model; (B) Pooled odds ratio for association of Pre-S2 deletion and HCC risk using random-effects model.

For Pre-S mutation, we primarily focused on Pre-S1 deletion and Pre-S2 deletion. 11 articles including 807 HCC cases and 1820 controls were included for Pre-S1 deletion and no significant heterogeneity was observed (I^2^ = 3.9%, P = 0.411). Thus fixed-effects model was used to appraise the association between Pre-S1 deletion and HCC. Significant correlation was obtained (OR = 2.94, 95%CI = 2.22–3.89) (See Figure 5A). For Pre-S2 deletion, 13 articles including 690 cases and 1140 controls were included. Heterogeneity seemed to exist (I^2^ = 43.3%) and random-effects model was used. The results indicated a significant correlation existed (OR = 3.02, 95% CI = 2.03–4.50) (See Figure 5B). Galbraith plot was used to determine the heterogeneity source and found Chen CH [21] was the sole outlier (See Figure 3B). After omitting that, significant correlation with no heterogeneity was observed as well (OR = 3.51, 95%CI = 2.61–4.73, I^2^ = 16.8%).

### The other common mutations and HCC risk

In our study, we appraised the correlation between the other common mutations like BCP double mutation, C1653T etc. For these mutations, statistically significant heterogeneity was observed for BCP double mutation (I^2^ = 76.1%, p<0.001), T1753V (I^2^ = 71.4%, p<0.001) and C1653T (I^2^ = 60.8%, p<0.001). Thus, for these mutations, random-effects model was used. Results showed that BCP double mutation A1762T/G1764A strongly correlated with the occurrence of HCC (OR = 3.98, 95%CI = 3.19–4.95) (See Figure S1); T1753V associated with a 2.23-fold risk (95% CI = 1.69–2.93) (See Figure S2), and C1653T with a 2.55-fold increased risk of HCC (95%CI = 1.95–3.35) (See Table S2, Figure S3).

In subgroup analysis by ethnicity, all the mutations correlated with the risk of HCC in Asians (For A1762T/G1764A, OR = 4.20, p<0.001; T1753V, OR = 2.19, p<0.001; C1653T, OR = 2.61, p<0.001). In subgroup analysis by genotype, A1762T/G1764A in HBV genotype B and C, T1753V in HBV genotype C and D, C1653T in genotype C patients significantly correlated with the increased risk of HCC. While in HBeAg subgroup, BCP A1762T/G1764A was associated with the increased risk of HCC irrespective of the HBeAg status. But in HBeAg positive group, BCP double mutation correlated with a higher risk than in negative group. While for C1653T, a higher risk susceptible to HCC could be seen in the negative group (See Table S2).

Besides, during the process of extracting data, we found an escalating trend of the mutant rate accompanying the progression of the disease and almost no difference could be observed between liver cirrhosis group and HCC group [4]. Therefore we categorized the four stages of disease state from asymptomatic carrier to HCC into two groups: HCC and liver cirrhosis as the progressive disease group, asymptomatic and chronic carrier as the non-progressive disease group. Significant correlations of the mutations with the progression of disease were found. BCP double mutation had the strongest association with the progressive disease group (summary OR = 5.46, 95%CI: 3.88–7.67). The odds ratio for T1753V was 2.77 (95%CI = 1.92–4.01), for C1653T was 2.59 (95%CI = 1.53–4.37). Moreover, we still observed significant correlation between G1896A and the progressive disease group with an OR = 1.85 (95%CI = 1.27–2.70) (See Table S2).

### Publication bias

**Figure 6:**
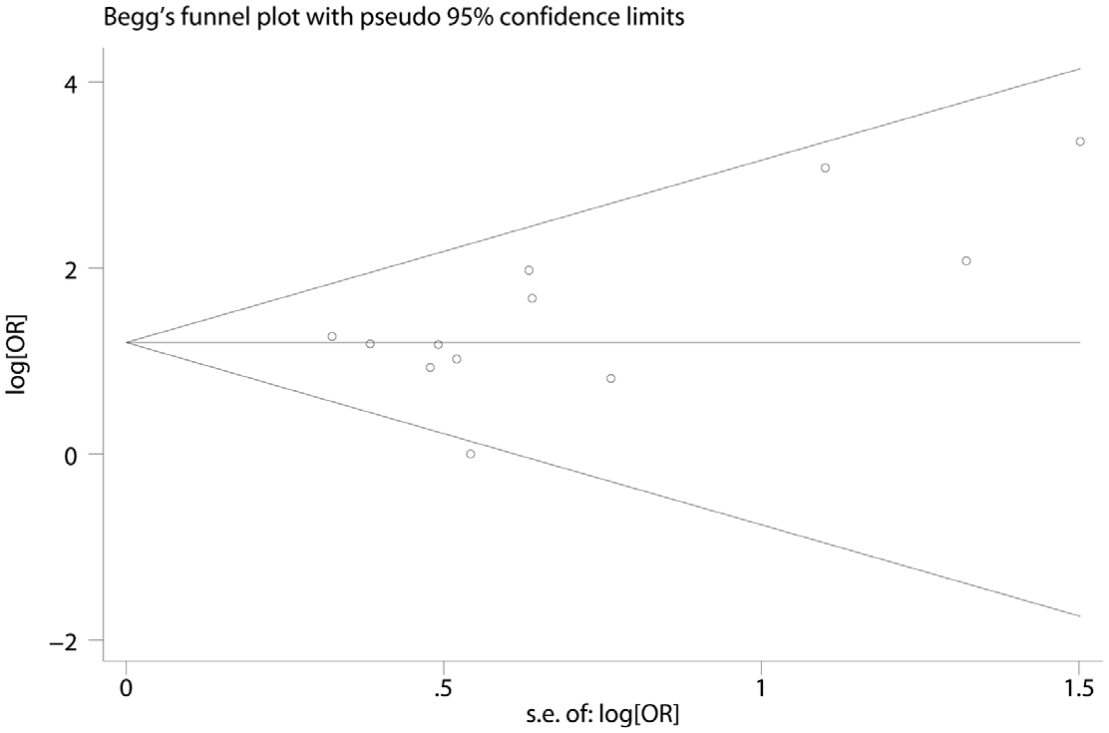
Funnel plot of association between Pre-S2 deletion and HCC risk (After adjustment for heterogeneity).

Publication bias was only found in Pre-S2 deletion through egger's test (P = 0.033). From Galbraith plot, we saw Chen CH [21] was the outlier. After omitting this study, heterogeneity greatly decreased from 43.3% to 16.8%. Then we reappraised the publication bias of Pre-S2 deletion and found no bias existed through egger's test (p = 0.113). The Begg's funnel plot seemed symmetrical as well (See Figure 6). No publication bias was found among the other mutations (See Table 1).

### Sensitivity analysis

For all the mutations, sensitivity analysis was performed by sequential omission of every study respectively. For every mutation, the odds ratio was not significantly influenced by omitting any single study (See Table S3).

## Discussion

Our study showed that Precore mutation G1896A and G1899A, Pre-S1 deletion, Pre-S2 deletion and other common mutations mentioned above were all independently associated with an increased risk of HCC as well as the progression of liver diseases.

Mutation G1896A locates at nt.1896 in the precore region leading to a G-to-A shift, which could induce a stop codon and subsequently suppress the expression of HBeAg [42]. Though it would cease the production of HBeAg, the HBV DNA would still be synthesized in patients and eventually contribute to the progression of liver disease to a more advanced stage [10]. It was once reported that G1896A correlated with severe forms of liver diseases [43]. Yin and his colleagues who did the former meta-analysis [16] also reported a significant association between G1896A and HCC with an OR = 5.10 in genotype B and 3.60 in genotype C patients in their recent research [4]. In our study, when pooling data from 43 studies including 3097 cases and 5530 controls, statistically significant association was determined between G1896A (OR = 1.46; p = 0.002) and the increased risk of HCC, which was totally different from Yin's [16] conclusion whose summary OR was 1.15 (95%CI = 0.83–1.60), pooling data from 24 studies. The discrepancy between our study and the previous one would attribute to the statistically power of detecting the slight difference since we included much more studies. The results after omitting the outliers and the sensitivity analysis again consolidated what we have gleaned. In subgroup analysis, we found correlation existed among Asians, but not in Caucasians or Africans. That might be due to insufficient studies focusing on the latter two populations. For genotype and HBeAg status subgroup, no definite conclusion could be drawn inasmuch as the insufficient studies, but the trend toward increasing the risk of HCC became obvious. Thus large-number, well-designed case-control studies are entailed in elucidating the relationship between G1896A and HCC risk.

Besides, G1899A also locates at Precore region of HBV genome and associated with a 3.14-fold increased risk of HCC compared with HBV patients without G1899A. For the mechanisms that underlie the interaction between G1899A and the onset of HCC still remain elusive. They may act as G1896A does but still merit further exploration. So far, this study is the first to report that significant correlation exists between G1899A and the increased risk of HCC based on a powerful meta-analysis, which might be a potential biomarker for foreseeing the occurrence of HCC.

Pre-S1 deletion and Pre-S2 deletion are the two most commonly seen mutations in Pre-S region. From our study, both types of mutations significantly correlated with the increased risk of HCC (For Pre-S1: OR = 2.94, p<0.001; Pre-S2: OR = 3.02, p<0.001). The deletion in Pre-S region would cause inefficient immune response and the pathogenesis of hepatocytes [43] and thus lead to the development of HCC. Given the significant association with HCC, the two mutations might be independently used as the biomarker for predicting the occurrence of HCC.

For the other common mutations, BCP double mutation A1762T/G1764A is generally accepted that it independently correlates with the risk of HCC and the progression of liver diseases [44], which is consistent with our results. For C1653T and T1753V, significant correlations with HCC are still observed in the two mutations. Though from Galbraith plot, we observed at least five outliers for each mutation (See Figure S4). This time we did not adjust the heterogeneity and reappraise the correlation since they have been generally accepted as the risk factors. Additionally, the sensitivity analysis confirmed that these mutations did strongly correlate with the risk of HCC. Thus our results are trustful.

From subgroup analysis by ethnicity, we could see that all the mutations mentioned in our study correlated with HCC among Asians, which might indicate that Asians would be more vulnerable to HCC. While for the relationship between genotypes and HCC, though the role of genotype in hepatocarcinogenesis remains elusive, several studies conducted in East Asia have proved that genotype C was more likely to cause severe liver disease like HCC than genotype B [45]–[47]. From our subgroup analysis, we may conclude that HBV genotype C patients with certain mutations like BCP double mutation, C1653T and T1753V would be exposed to a much higher risk of HCC. While genotype B has a moderate effect with such mutation on HCC compared to genotype C which is consistent with those previous studies. When stratified by HBeAg statuses, BCP double mutation and positive HBeAg status may have synergistic effect on the occurrence of HCC. For the other mutations, more studies are necessitated to explore the impact these mutations have on patients with different HBeAg statuses.

For the four stages of liver diseases, when we divided them into two groups: HCC and LC; chronic hepatitis and asymptomatic carrier, we found that the higher the incidence of HBV mutations, the more severe the liver disease. We found a 5.46-fold increased risk of HCC and LC in BCP double mutation, 2.77-fold in T1753V and 2.59-fold in C1653T. Precore G1896A was still associated with the progressive diseases contributing to a 1.85-fold increased risk suffering from a more severe disease status. Besides, Yin and his colleagues [4] also reported the correlation between these mutations and the liver disease progression, which coincided with our conclusion. Based on these, we may infer that these mutations would have accumulative effects in the development of the liver diseases and therefore a higher frequency of occurrence could be observed in HCC and LC. Meanwhile, their roles in promoting the development of a severer liver disease status could in part explain their associations with HCC onset. Thus these mutations may additionally contribute to the progression of liver disease, apart from their influences on the occurrence of HCC.

However, our study has several limitations. Firstly, a large number of studies were included in this study, and inevitably there existed heterogeneity which could not be easily eliminated. Thus we adopted random-effects model. When doing the sensitivity analysis, the odds ratios were not significantly affected. Therefore it should not influence the accuracy of our results. Secondly, when adjusting for heterogeneity, we excluded those outliers seen from Galbraith plot and during the excluding process, extra bias might be introduced. Thirdly, when taking subgroup analysis, only a few studies were available and the evidence seemed insufficient as well as unconvincing. Fourthly, since few studies involved the evaluation of the combination effects of these mutations on HCC, we did not summarize the total effects for the combination of these mutations. Lastly, only the data of those published articles in English or Chinese can be extracted and a potential bias would thus be introduced. Given these limitations, what we have found in this study should be interpreted prudently.

Despite these limitations, our study indicates that Precore mutation G1896A is significantly associated with the increased risk of HCC and the progression of liver disease, especially among Asians. Besides, G1899A, Pre-S1 deletion, Pre-S2 deletion as well as other common mutations like BCP double mutation A1762T/G1764A, T1753V and C1653T, all of them correlate with HCC risk. For those results in subgroup analysis, they still need large-scale, well-designed case-control studies to prove. Since the development of the hepatocellular carcinoma can be attributed to multiple factors, what we have observed is only the tip of the iceberg. The future study could concentrate on the interaction of epigenetic influence as well as the genetic variations. Certainly, the environment and the host factors should be taken into consideration.

## Supporting Information

Figure S1 **Forest plot for correlation of BCP double mutation A1762T/G1764A and HCC risk.**
(TIF)

Figure S2 **Forest plot for correlation of T1753V and HCC risk.**
(TIF)

Figure S3 **Forest plot for correlation of C1653T and HCC risk.**
(TIF)

Figure S4 Galbraith plot for heterogeneity of BCP double mutation, T1753V and C1653T (A: BCP double mutatin; B: T1753V; C: C1653T).
(TIF)

Table S1 Characteristics of eligible studies included in the meta-analysis.
(DOC)

Table S2 Odds ratio for correlation of the other common mutations with HCC risk in the meta-analysis.
(DOC)

Table S3 Sensitivity analysis of each mutation.
(XLS)
