# Supplementary figures and images for: Precore Mutation of Hepatitis B Virus May Contribute to Hepatocellular Carcinoma Risk: Evidence from an Updated Meta-Analysis
Source: PLoS One. 2012 Jun 1;7(6):e38394. doi: 10.1371/journal.pone.0038394 (PMC3365888; doi:10.1371/journal.pone.0038394)

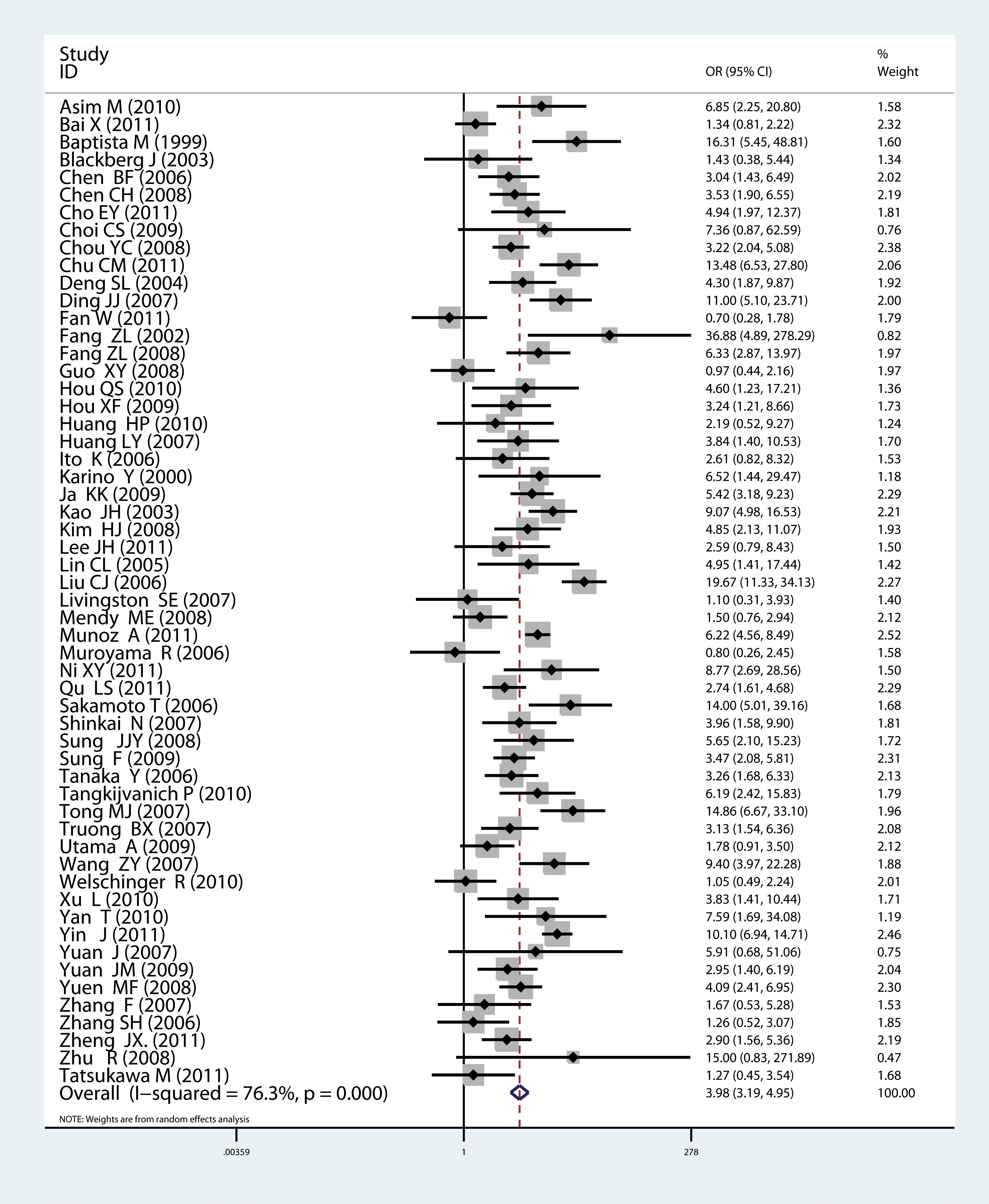

Supplement: Figure S1 — Forest plot for correlation of BCP double mutation A1762T/G1764A and HCC risk. (TIF) [file pone.0038394.s001.tif]

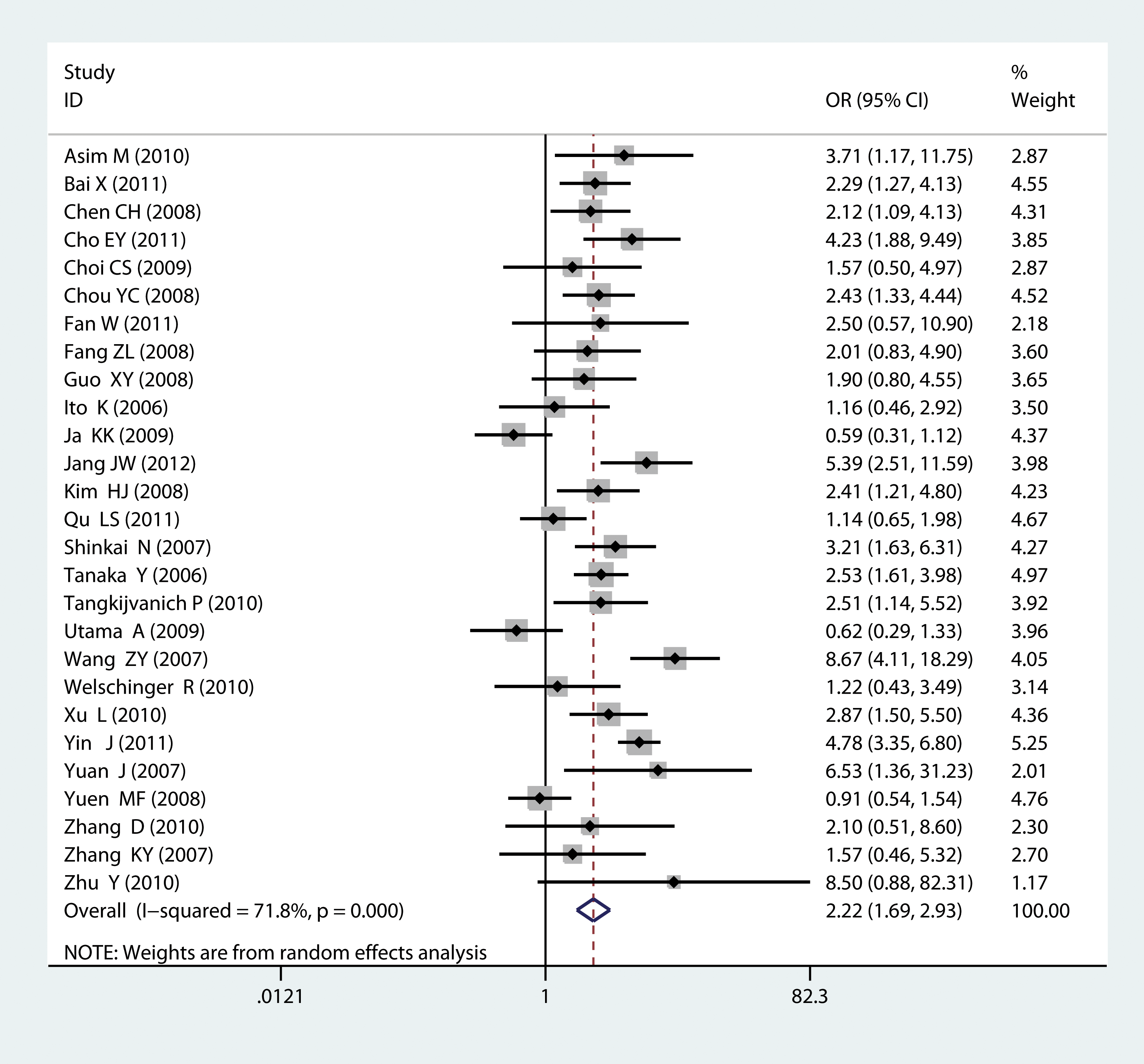

Supplement: Figure S2 — Forest plot for correlation of T1753V and HCC risk. (TIF) [file pone.0038394.s002.tif]

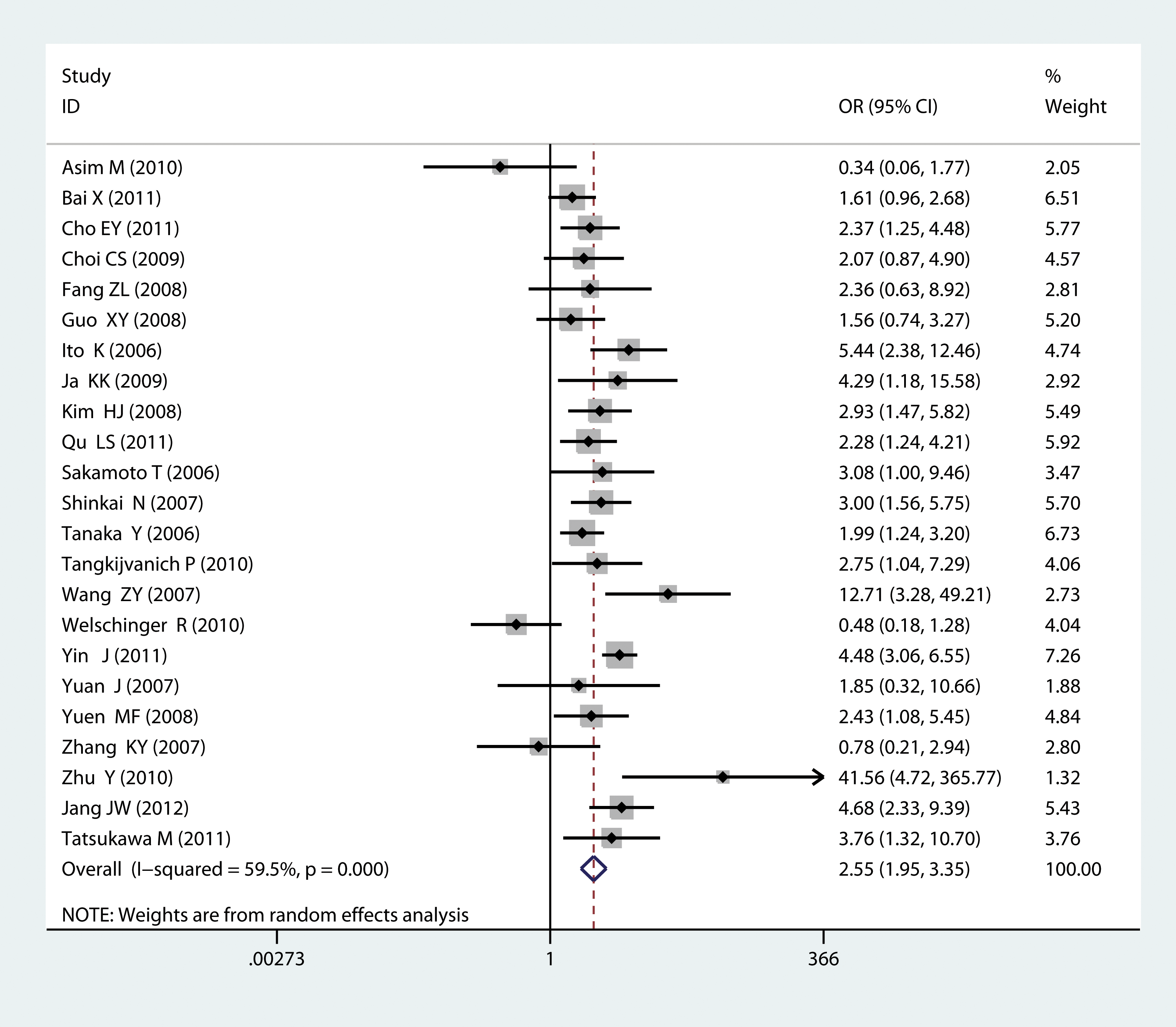

Supplement: Figure S3 — Forest plot for correlation of C1653T and HCC risk. (TIF) [file pone.0038394.s003.tif]

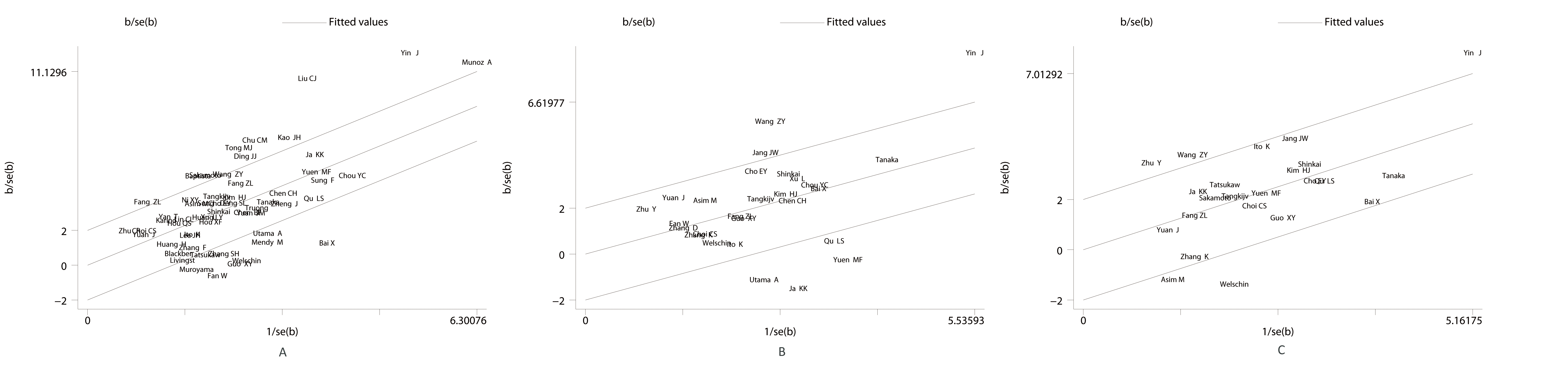

Supplement: Figure S4 — Galbraith plot for heterogeneity of BCP double mutation, T1753V and C1653T (A: BCP double mutatin; B: T1753V; C: C1653T). (TIF) [file pone.0038394.s004.tif]
