# Supplementary material for: Precore Mutation of Hepatitis B Virus May Contribute to Hepatocellular Carcinoma Risk: Evidence from an Updated Meta-Analysis
Source: PLoS One. 2012 Jun 1;7(6):e38394. doi: 10.1371/journal.pone.0038394 (PMC3365888; doi:10.1371/journal.pone.0038394)
Supplement: Table S1 — Characteristics of eligible studies included in the meta-analysis. (DOC) [file pone.0038394.s005.doc]

| Table S1. Characteristics of eligible studies included in the meta-analysis | | | | | | | | | | | |
| --- | --- | --- | --- | --- | --- | --- | --- | --- | --- | --- | --- |
| Author | Year | Country | Ethnicity | Design | Detection method | No. of case/control | Mean age(±SD)(year) | Sex (male%) | | HBV genotype | Mutation site |
|  |  |  |  |  |  |  |  | Control | Case |  |  |
| Asim M | 2010 | India | Asian | PCC | nested-PCR | 39/29 | 53.6±13.2(case), 44.2±14.7(control) | - | - | A,D | T1762/A1764,T1753V,A1653T,G1899A,G1896A |
| Bai X | 2011 | China | Asian | PCC | sequence | 152/136 | 47.9±11.9(case), 45.6 ± 11.4(control) | 89.7 | 85.5 | B,C | A1762T/G1764A,T1753, A1653,A1762,T1764 |
| Baptista M | 1999 | South Africa | African | PCC | sequence | 50/47 | - | - | - | - | A1762T/G1764A,T1764 |
| Blackberg J | 2003 | Mixed | Mixed | PCC | sequence | 16/19 | 61(case),55(control) | **-** | - | A,B,C,D | Pre-S,A1762T/G1764A,G1896A,T1762,T1764 |
| Cao Z | 2008 | China | Asian | NCC | PAGE | 47/50 | 42.1 ± 8.8(case); 39.9 ± 12.4(control) | 94.0 | 95.7 | - | Pre-S |
| Chen BF | 2006 | Taiwan | Asian | PCC | INNO-LiPA | 50/102 | 45.2±7.6(case) | 66.7 | 88.0 | B,C | Pre-S,A1762T/G1764A,G1896A |
| Chen CH | 2008 | Taiwan | Asian | NCC | sequence | 80/160 | 49.5±10.8 | 82.5 | 82.5 | B,C,D | Pre-S,T1762/A1764,G1896A,G1899A |
| Cho EY | 2011 | Korea | Asian | ICC | sequence | 69/125 | - | 69.6 | 68.1 | - | A1762T/G1764A,T1753V,C1653T |
| Choi MS | 2007 | Korea | Asian | PCC | sequence | 72/228 | 51.9 ± 7.8(case) | 71.9 | 80.6 | C | Pre-S |
| Choi CS | 2009 | Korea | Asian | PCC | sequence | 42/46 | 57.3±9.3(case) 56.0±8.5(control) | 73.8 | 56.5 | C | T1762/A1764,T1753V,C1653T |
| Chou YC | 2008 | Taiwan | Asian | NCC | sequence | 132/204 | - | - | - | B,C | T1762/A1764,G1899A,T1753V |
| Chu CM | 2011 | Taiwan | Asian | NCC | PCR-RFLP | 125/80 | - | 78.1 | 36.4 | B,C | T1762/A1763,G1896A |
| Deng SL | 2004 | China | Asian | PCC | Hybridization+Elisa | 114/100 | - | - | - | - | T1762/A1764 |
| Ding JJ | 2007 | China | Asian | PCC | PCR-RFLP | 42/442 | 30.56±23.62 | - | - | - | T1762/A1764,G1896A |
| Fan W | 2011 | China | Asian | PCC | sequence | 34/38 | - | 65.8 | 79.4 | B.C | T1762/A1764,T1753V |
| Fang ZL | 2008 | China | Asian | PCC | sequence | 33/33 | - | 75.8 | 75.8 | - | Pre-S |
| Fang ZL | 2002 | China | Asian | ICC | sequence | 36/115 | - | - | - | B,C | T1762/A1764,G1896A |
| Fang ZL | 2008 | China | Asian | PCC | sequence | 30/30 | - | - | - | - | T1762/A1764,T1753V,C1653T |
| Gao ZY | 2007 | China | Asian | PCC | sequence | 26/53 | 55.56 ± 12.28(case) | 56.6 | 88.5 | C | Pre-S |
| Guo XY | 2008 | China | Asian | NCC | nested-PCR | 58/71 | 41.9 ± 10.4(case), 39.9 ± 9.1(control) | - | - | - | T1762/A1764,T1753V,C1653T,  T1762,T1764 |
| Hou QS | 2010 | China | Asian | PCC | RT-PCR | 26/56 | - | - | - | - | T1762/A1764,G1896A |
| Hou XF | 2009 | China | Asian | PCC | sequence | 34/83 | 43.5±12.6 | - | - | B,C | T1762/A1764,G1896A |
| Huang H | 2010 | Taiwan | Asian | NCC | sequence | 19/19 | 12.7±6.6(case), 12.3±0.8(control) | 68.4 | 68.4 | B,C | Pre-S,T1762/A1764,G1896A |
| Huang L | 2007 | China | Asian | PCC | sequence | 20/156 | 38.6±11.4 | - | - | - | T1762/A1764 |
| Huy TT | 2003 | Mixed | mixed | PCC | sequence | 49/264 | - | - | - | - | Pre-S |
| Ito K | 2006 | Japan | Asian | ICC | sequence | 40/80 | 50.7± 9.4(case) | 85.0 | 90.0 | C | T1762/A1764,G1896A,T1753V,C1653T,C1858T |
| Ja KK | 2009 | Korea | Asian | PCC | sequence | 135/135 | 44.3±7.8(case) | - | - | C | T1762/A1764,G1896A,T1753V,C1653T,C1858T |
| Jang JS | 2009 | Korea | Asian | PCC | sequence | 48/71 | - | - | - | C | Pre-S |
| Jang JW | 2012 | Korea | Asian | ICC | sequence | 75/75 | - | 78.7 | 78.7 | C | T1762,A1764,G1896A,T1753V,C1653T,G1899A |
| Kao JH | 2003 | Taiwan | Asian | NCC | sequence | 127/123 | - | - | - | B,C | T1762/A1764,G1896A |
| Karino Y | 2000 | Japan | Asian | PCC | Sequence | 32/110 | - | - | - | - | T1762/A1764A1897 |
| Kim HJ | 2008 | Korea | Asian | PCC | Sequence | 60/124 | 45.9±17.3 | - | - | C | T1762/A1764,T1753V,A1653T |
| Laskus T | 1998 | Gambia | African | ICC | Sequence | 27/33 | 45.4±9.2(case), 47.5±10.2(control) | 85.0 | 82.0 | - | G1896A,G1899A |
| Lee JH | 2011 | Korea | Asian | PCC | Sequence | 31/65 | 55.6±7.8(case) | 67.7 | 83.9 | - | T1762/A1764 |
| Lee MH | 2011 | Korea | Asian | NCC | Sequence | 135/135 | 44.3±7.8(case), 44.3±8.0(control) | 83.0 | 83.0 | C | Pre-S,G1896A |
| Li HF | 2011 | China | Asian | PCC | Sequence | 26/60 | 58.54±8.46(case) | - | - | B,C | Pre-S |
| Lin CL | 2005 | Taiwan | Asian | ICC | Sequence | 32/142 | 58±13(case) | - | - | B,C | T1762/A1764,G1896A |
| Lin CL | 2007 | Taiwan | Asian | ICC | INNO-LiPA | 64/202 | 55±12(case) | 66.8 | 89.0 | B,C | Pre-S |
| Liu CJ | 2006 | Taiwan | Asian | PCC | INNO-LiPA | 199/159 | 54.0±12.3(case) | 38.2 | 61.9 | B,C | T1762/A1764,G1896A |
| Liu N | 1998 | China | Asian | ICC | PCR-RFLP | 83/66 | 51.39±12.14(case) | - | - | - | G1896A |
| Livingston SE | 2007 | USA | America | ICC | sequence | 33/31 | - | - | - | A,C,D,F | T1762/A1764,G1896A |
| Mendy M | 2008 | Gambia | African | PCC | sequence | 119/64 | - | - | - | - | T1762/A1764,G1896A |
| Mun HS | 2011 | Korea | Asian | PCC | sequence | 99/142 | 56.4±10.9(case) | 82.4 | 85.5 | C | Pre-S_2_ |
| Mun HS | 2008 | Korea | Asian | PCC | sequence | 40/80 | 47.9±17.3(total) | - | - | - | Pre-S |
| Munoz A | 2011 | China | Asian | NCC | RT-PCR | 345/625 | - | - | - | - | T1762/A1764 |
| Muroyama R | 2006 | Japan | Asian | PCC | sequence | 39/36 | 56.5 ± 10(case), 54.5 ± 9(control) | 94.0 | 95.0 | C | T1762/A1764,G1896A |
| Ni Y | 2003 | Taiwan | Asian | ICC | sequence | 12/23 | - | 54.8 | 75.0 | B | G1896A |
| Ni XY | 2011 | China | Asian | PCC | sequence | 35/32 | - | - | - | - | T1762/A1764 |
| Qiang FL | 2011 | China | Asian | ICC | sequence | 26/25 | 69.29±42.17(case) 19.43±4.46(control) | - | - | - | Pre-S |
| Qu LS | 2011 | China | Asian | ICC | sequence | 134/114 | - | - | - | B,C | Pre-S,T1762/A1764,G1896A,G1899A,T1753V,C1653T |
| Raimondo G | 2004 | Italy | Europe | PCC | sequence | 19/90 | 60(case) | 71.1 | 89.5 | - | Pre-S |
| Sakamoto T | 2006 | Philippine | Asian | ICC | sequence | 31/69 | 53.7±15.1(case) | 72.5 | 87.0 | A,B,C | T1762/A1764,G1896A,C1653T  C1858T,T1809 |
| Shinkai N | 2007 | Japan | Asian | PCC | sequence | 80/80 | 55±8(case), 54±8(control) | 79.8 | 80.0 | C | T1762/A1764,G1896A,T1753V  C1653T,T1479C |
| Sugauchi F | 2003 | Japan | Asian | PCC | sequence | 24/136 | - | - | - | B,C | Pre-S |
| Sung JJ | 2008 | Hongkong | Asian | ICC | seminested PCR | 100/100 | - | 73.0 | 87.0 | B,C | T1762/A1764,G1899A |
| Sung F | 2009 | Taiwan | Asian | NCC | sequence | 116/145 | 50.1±8.9(case), 50.6±9.0(control) | - | - | A,B,C | G1896A, T1762/A1764 |
| Tanaka Y | 2006 | Japan | Asian | PCC | sequence | 148/180 | - | - | - | C | T1762/A1764,G1896A,  T1753V,C1653T |
| Tangkijvanich P | 2010 | Thailand | Asian | PCC | sequence | 60/60 | 55.7 ± 9.8(case), 52.9±8.6(control) | 86.7 | 86.7 | B,C | T1762/A1764,G1896A,G1899A,  T1753V,C1653T |
| Tong MJ | 2007 | USA | mixed | ICC | sequence | 101/67 | 53.3±13.5(case), 45.4±12.3(control) | 43.3 | 83.0 | A,B,C,D | T1762/A1764,G1896A |
| Tatsukawa M | 2011 | Japan | Asian | ICC | sequence | 37/38 | 37 ± 10.0(case), 38 ± 11.6(control) | 73.0 | 78.0 | C | T1762/A1764,C1653T |
| Truong B | 2007 | Vietnam | Asian | PCC | sequence | 48/135 | 39.5±16.5(total) | 80 | 95.8 | B,C | T1762/A1764,G1896A,C1858T |
| Utama A | 2009 | Indonesia | Asian | PCC | sequence | 36/90 | 49.6 ± 10.4 (case) | 68.3 | 89.6 | B,C | T1762/A1764,T1753,T1762,T1764 |
| Utama A | 2011 | Indonesia | Asian | PCC | sequence | 62/203 | 42.8±13.2(total) | 79.8 | 77.4 | B,C,D | Pre-S |
| Wang HC | 2006 | Taiwan | Asian | NCC | sequence | 68/132 | - | - | - | - | Pre-S |
| Wang Y | 2000 | China | Asian | PCC | sequence | 23/72 | - | - | - | - | G1896A, |
| Wang ZY | 2007 | China | Asian | PCC | sequence | 47/164 | 49.8±11.6(case) | 81.7 | 89.4 | B,C | T1762/A1764,G1896A,G1899A,T1753V,C1653T,C1858T |
| Wang XF | 1996 | China | Asian | ICC | - | - | - | - | - | - | G1896A |
| Wang XY | 2010 | China | Asian | NCC | sequence | 33/33 | - | 75.8 | 75.8 | - | Pre-S |
| Welschinger R | 2010 | South Africa | African | PCC | sequence | 84/50 | - | - | - | A,B,C,D,F | T1762/A1764,T1753V,C1653T,T1762,T1764 |
| Xu L | 2010 | China | Asian | NCC | sequence | 60/120 | 41 ± 9(case), 42 ± 7(control) | 90.0 | 90.0 | B,C | T1762/A1764,T1753V |
| Yan T | 2010 | China | Asian | PCC | sequence | 27/90 | 52.6±10.5(case) | 77.8 | 77.8 | B,C | T1762/A1764 |
| Yeung P | 2011 | Hongkong | Asian | NCC | sequence | 96/96 | 56.9±11.5(case), 56.8±11.5(control) | 85.4 | 85.4 | B,C | Pre-S |
| Yin J | 2011 | China | Asian | ICC | sequence | 190/1269 | 50.2±11.0(case) | 62.9 | 79.5 | B,C | T1762/A1764,T1753V,C1653T,C1673T |
| Yin J | 2010 | China | Asian | ICC | sequence | 231/941 | 49.9 ± 11.0(case) | 62.7 | 77.5 | B,C | Pre-S |
| Yuan J | 2007 | China | Asian | PCC | sequence | 8/59 | 49.53±12.86(case) | - | - | B,C | T1762/A1764,G1896A,G1899A,T1753V,C1653T,C1858T |
| Yuan JM | 2009 | China | Asian | PCC | RT-PCR | 49/97 | 57.98±4.23(case), | - | - | - | T1762/A1764, |
| Yuen MF | 2004 | Hongkong | Asian | ICC | sequence | 66/135 | 61.1(total) |  |  | B,C | T1762/A1764,A1896 |
| Yuen MF | 2008 | Hongkong | Asian | ICC | sequence | 248/248 | 57.5(case), 57.7(control) | 80.2 | 80.2 | B,C,D | T1762/A1764,G1896A,T1753V,C1653T |
| Zhang D | 2010 | China | Asian | PCC | sequence | 11/132 | 38 ± 13(total) | 71.2 | 90.9 | B,C | G1896A,T1753V,T1762,T1764 |
| Zhang F | 2007 | China | Asian | NCC | sequence | 28/28 | - | - | - | - | T1762/A1764, |
| Zhang K | 2007 | Japan | Asian | PCC | sequence | 24/20 | 47.33 ± 9.14(case) | 70.0 | 87.5 | B,C | G1896A,T1753V,C1653T |
| Zhang S | 2006 | China | Asian | PCC | Hybridization+Elisa | 30/57 | - | - | - | - | T1762/A1764 |
| Zheng J | 2011 | China | Asian | PCC | sequence | 156/185 | - | 75.7 | 83.3 | - | T1762/A1764,G1896A,G1899A |
| Zhu R | 2008 | China | Asian | NCC | sequence | 7/76 | 42.71±9.23(case) | 86.8 | 100.0 | C | T1762/A1764 |
| Zhu Y | 2010 | China | Asian | PCC | sequence | 20/35 | - | - | - | B,C | G1896A,G1899A,T1753V,C1653T,T1762, |

- HBV= Hepatitis B virus; INNO-Lipa= Innogenetics line probe assay ; PCR= polymorphism chain reaction ; ELISA= enzyme linked immunosorbent assay ; RFLP= restricted fragment length porlymorphism ; PAGE=polyacrylamide gel electrophoresis ; PCC= prevalence case-control ; ICC= Incidence case-control ; NCC= nested case-control.

**Reference :**

1. Asim M, Malik A, Sarma MP, Polipalli SK, Begum N, et al. (2010) Hepatitis B virus BCP, Precore/core, X gene mutations/genotypes and the risk of hepatocellular carcinoma in India. J Med Virol 82: 1115-1125.

2. Bai X, Zhu Y, Jin Y, Guo X, Qian G, et al. (2011) Temporal acquisition of sequential mutations in the enhancer II and basal core promoter of HBV in individuals at high risk for hepatocellular carcinoma. Carcinogenesis 32: 63-68.

3. Baptista M, Kramvis A, Kew MC (1999) High prevalence of 1762(T) 1764(A) mutations in the basic core promoter of hepatitis B virus isolated from black Africans with hepatocellular carcinoma compared with asymptomatic carriers. Hepatology 29: 946-953.

4. Blackberg J, Kidd-Ljunggren K (2003) Mutations within the hepatitis B virus genome among chronic hepatitis B patients with hepatocellular carcinoma. J Med Virol 71: 18-23.

5. Cao Z, Bai X, Guo X, Jin Y, Qian G, et al. (2008) High prevalence of hepatitis B virus pre-S mutation and its association with hepatocellular carcinoma in Qidong, China. Arch Virol 153: 1807-1812.

6. Chen BF, Liu CJ, Jow GM, Chen PJ, Kao JH, et al. (2006) High prevalence and mapping of pre-S deletion in hepatitis B virus carriers with progressive liver diseases. Gastroenterology 130: 1153-1168.

7. Chen CH, Changchien CS, Lee CM, Hung CH, Hu TH, et al. (2008) Combined mutations in pre-s/surface and core promoter/precore regions of hepatitis B virus increase the risk of hepatocellular carcinoma: a case-control study. J Infect Dis 198: 1634-1642.

8. Cho EY, Choi CS, Cho JH, Kim HC (2011) Association between Hepatitis B Virus X Gene Mutations and Clinical Status in Patients with Chronic Hepatitis B Infection. Gut Liver 5: 70-76.

9. Choi MS, Kim DY, Lee DH, Lee JH, Koh KC, et al. (2007) Clinical significance of pre-S mutations in patients with genotype C hepatitis B virus infection. J Viral Hepat 14: 161-168.

10. Choi CS, Cho EY, Park R, Kim SJ, Cho JH, et al. (2009) X gene mutations in hepatitis B patients with cirrhosis, with and without hepatocellular carcinoma. J Med Virol 81: 1721-1725.

11. Chou YC, Yu MW, Wu CF, Yang SY, Lin CL, et al. (2008) Temporal relationship between hepatitis B virus enhancer II/basal core promoter sequence variation and risk of hepatocellular carcinoma. Gut 57: 91.

12. Chu CM, Lin CC, Lin SM, Lin DY, Liaw YF (2011) Viral Load, Genotypes, and Mutants in Hepatitis B Virus-Related Hepatocellular Carcinoma: Special Emphasis on Patients with Early Hepatocellular Carcinoma. Dig Dis Sci.

13. Deng S (2004) Study on HBV-DNA concentration and HBV C gene promotor gene mutation of hepatoma patients[Chinese]. Chongqing Medical Journal 6: 880-881.

14. Ding JJ, Liu YH, Wang M (2007) [Study on the distribution of hepatitis B virus precore and basic core promoter mutations in Guizhou area]. Zhonghua Liu Xing Bing Xue Za Zhi 28: 169-172.

15. Fan W, Shi B, Wei H, Du G, Song S (2011) Comparison of hepatitis B X gene mutation between patients with hepatocellular carcinoma and patients with chronic hepatitis B. Virus Genes 42: 162-170.

16. Fang ZL, Sabin CA, Dong BQ, Wei SC, Chen QY, et al. (2008) Hepatitis B virus pre-S deletion mutations are a risk factor for hepatocellular carcinoma: a matched nested case-control study. J Gen Virol 89: 2882-2890.

17. Fang ZL, Yang J, Ge X, Zhuang H, Gong J, et al. (2002) Core promoter mutations (A1762T and G1764A) and viral genotype in chronic hepatitis B and hepatocellular carcinoma in Guangxi, China. Journal of Medical Virology 68: 33-40.

18. Fang ZL, Sabin CA, Dong BQ, Ge LY, Wei SC, et al. (2008) HBV A1762T, G1764A mutations are a valuable biomarker for identifying a subset of male HBsAg carriers at extremely high risk of hepatocellular carcinoma: a prospective study. Am J Gastroenterol 103: 2254-2262.

19. Gao ZY, Li T, Wang J, Du JM, Li YJ, et al. (2007) Mutations in preS genes of genotype C hepatitis B virus in patients with chronic hepatitis B and hepatocellular carcinoma. Journal of Gastroenterology.

20. Guo X, Jin Y, Qian G, Tu H (2008) Sequential accumulation of the mutations in core promoter of hepatitis B virus is associated with the development of hepatocellular carcinoma in Qidong, China. J Hepatol 49: 718-725.

21. Hou Q (2010) Study on the relationship of multi-mutation of hepatitis B virus gene with hepatocellular carcinoma[Chinese]. National Medical Frontiers of China 01: 15-16.

22. Hou X (2009) Mutations in the core promoter of hepatitis B virus and viral genotype are correlated with hepatocellular carcinoma. Hainan Medical Journal 02: 1-3.

23. Huang HP, Hsu HY, Chen CL, Ni YH, Wang HY, et al. (2010) Pre-S2 deletions of hepatitis B virus and hepatocellular carcinoma in children. Pediatric Research 67: 90-94.

24. Huang L (2007) Relationship between HBV BCP mutation and serum IL-10, IL-12, TNF-α as well as IFN-γ in HCC[Chinese]. China Journal of Modern Medicine 20: 2488-2491.

25. Huy TT, Ushijima H, Win KM, Luengrojanakul P, Shrestha PK, et al. (2003) High prevalence of hepatitis B virus pre-s mutant in countries where it is endemic and its relationship with genotype and chronicity. J Clin Microbiol 41: 5449-5455.

26. Ito K, Tanaka Y, Kato M, Fujiwara K, Sugauchi F, et al. (2007) Comparison of complete sequences of hepatitis B virus genotype C between inactive carriers and hepatocellular carcinoma patients before and after seroconversion. J Gastroenterol 42: 837-844.

27. Ja KK, Hye YC, Jung ML, Baatarkhuu O, Young JY, et al. (2009) Specific mutations in the enhancer II/core promoter/precore regions of hepatitis B virus subgenotype C2 in Korean patients with hepatocellular carcinoma. Journal of Medical Virology 81: 1002-1008.

28. Jang JS, Kim HS, Kim HJ, Shin WG, Kim KH, et al. (2009) Association of concurrent hepatitis B surface antigen and antibody to hepatitis B surface antigen with hepatocellular carcinoma in chronic hepatitis B virus infection. J Med Virol 81: 1531-1538.

29. Jang JW, Chun JY, Park YM, Shin SK, Yoo W, et al. (2012) Mutational complex genotype of the hepatitis B virus X /precore regions as a novel predictive marker for hepatocellular carcinoma. Cancer Sci 103: 296-304.

30. Kao JH, Chen PJ, Lai MY, Chen DS (2003) Basal core promoter mutations of hepatitis B virus increase the risk of hepatocellular carcinoma in hepatitis B carriers. Gastroenterology 124: 327-334.

31. Karino Y, Toyota J, Sato T, Ohmura T, Yamazaki K, et al. (2000) Early mutation of precore (A1896) region prior to core promoter region mutation leads to decrease of HBV replication and remission of hepatic inflammation. Dig Dis Sci 45: 2207-2213.

32. Kim HJ, Park JH, Jee Y, Lee SA, Kim H, et al. (2008) Hepatitis B virus X mutations occurring naturally associated with clinical severity of liver disease among Korean patients with chronic genotype C infection. J Med Virol 80: 1337-1343.

33. Laskus T, Radkowski M, Nowicki M, Wang LF, Vargas H, et al. (1998) Association between hepatitis B virus core promoter rearrangements and hepatocellular carcinoma. Biochem Biophys Res Commun 244: 812-814.

34. Lee JH, Han KH, Lee JM, Park JH, Kim HS (2011) Impact of hepatitis B virus (HBV) x gene mutations on hepatocellular carcinoma development in chronic HBV infection. Clin Vaccine Immunol 18: 914-921.

35. Lee MH, Kim DY, Kim JK, Chang HY, Kang SH, et al. (2011) Combination of preS Deletions and A1762T/G1764A Mutations in HBV Subgenotype C2 Increases the Risk of Developing HCC. Intervirology.

36. Li H, Qiang F, Yang Z, Spencer SD, Cui X, et al. (2011) Hepatitis B virus with pre-S2 deletion is more prevalent in hepatocellular carcinoma than in chronic active hepatitis and asymptomatic carriers. Acta Virol 55: 183-185.

37. Lin CL, Liao LY, Wang CS, Chen PJ, Lai MY, et al. (2005) Basal core-promoter mutant of hepatitis B virus and progression of liver disease in hepatitis B e antigen-negative chronic hepatitis B. Liver International 25: 564-570.

38. Lin CL, Liu CH, Chen W, Huang WL, Chen PJ, et al. (2007) Association of pre-S deletion mutant of hepatitis B virus with risk of hepatocellular carcinoma. J Gastroenterol Hepatol 22: 1098-1103.

39. Liu CJ, Chen BF, Chen PJ, Lai MY, Huang WL, et al. (2006) Role of hepatitis B viral load and basal core promoter mutation in hepatocellular carcinoma in hepatitis B carriers. J Infect Dis 193: 1258-1265.

40. Liu N (1998) Study on the HBV PreC A1896 mtation in the serum and the liver tissue of the parimary hepatocellular carcinoma patients. JIANGSU MEDICAL JOURNAL 12: 878-880.

41. Livingston SE, Simonetti JP, McMahon BJ, Bulkow LR, Hurlburt KJ, et al. (2007) Hepatitis B virus genotypes in Alaska Native people with hepatocellular carcinoma: preponderance of genotype F. J Infect Dis 195: 5-11.

42. Mendy ME, Kaye S, Le Roux E, Kirk GD, Jeng-Barry A, et al. (2008) Application of a novel, rapid, and sensitive oligonucleotide ligation assay for detection of cancer-predicting mutations in the precore and basal core promoter of hepatitis B virus. J Clin Microbiol 46: 2723-2730.

43. Mun HS, Lee SA, Kim H, Hwang ES, Kook YH, et al. (2011) Novel F141L pre-S2 mutation in hepatitis B virus increases the risk of hepatocellular carcinoma in patients with chronic genotype C infections. J Virol 85: 123-132.

44. Mun HS, Lee SA, Jee Y, Kim H, Park JH, et al. (2008) The prevalence of hepatitis B virus preS deletions occurring naturally in Korean patients infected chronically with genotype C. J Med Virol 80: 1189-1194.

45. Munoz A, Chen JG, Egner PA, Marshall ML, Johnson JL, et al. (2011) Predictive power of hepatitis B 1762T/1764A mutations in plasma for hepatocellular carcinoma risk in Qidong, China. Carcinogenesis 32: 860-865.

46. Muroyama R, Kato N, Yoshida H, Otsuka M, Moriyama M, et al. (2006) Nucleotide change of codon 38 in the X gene of hepatitis B virus genotype C is associated with an increased risk of hepatocellular carcinoma. J Hepatol 45: 805-812.

47. Ni Y, Chang M, Hsu H, Tsuei D (2003) Different hepatitis B virus core gene mutations in children with chronic infection and hepatocellular carcinoma. Gut 52: 122.

48. Ni X (2011) Associations between A1762T/G1764A mutations of hepatitis B virus and the risk of hepatocellular carcinoma[Chinese]. Chinese Journal of Current Advances in General Surgery 1: 59-61.

49. Qiang F (2011) Associations between Hepatitis B Virus PreS2 Deletion Mutation and Hepatocarcinoma[Chinese]. The Practical Journal of Cancer 1: 20-21.

50. Qu LS, Liu TT, Jin F, Guo YM, Chen TY, et al. (2011) Combined pre-S deletion and core promoter mutations related to hepatocellular carcinoma: A nested case-control study in China. Hepatol Res 41: 54-63.

51. Raimondo G (2004) Non-sequencing molecular approaches to identify preS2-defective hepatitis B virus variants proved to be associated with severe liver diseases. Journal of Hepatology 40: 515-519.

52. Sakamoto T, Tanaka Y, Orito E, Co J, Clavio J, et al. (2006) Novel subtypes (subgenotypes) of hepatitis B virus genotypes B and C among chronic liver disease patients in the Philippines. Journal of general virology 87: 1873.

53. Shinkai N, Tanaka Y, Ito K, Mukaide M, Hasegawa I, et al. (2007) Influence of hepatitis B virus X and core promoter mutations on hepatocellular carcinoma among patients infected with subgenotype C2. J Clin Microbiol 45: 3191-3197.

54. Sugauchi F, Ohno T, Orito E, Sakugawa H, Ichida T, et al. (2003) Influence of hepatitis B virus genotypes on the development of preS deletions and advanced liver disease. J Med Virol 70: 537-544.

55. Sung JJY, Tsui SKW, Tse CH, Ng EYT, Leung KS, et al. (2008) Genotype-specific genomic markers associated with primary hepatomas, based on complete genomic sequencing of hepatitis B virus. Journal of Virology 82: 3604-3611.

56. Sung F, Jung C, Wu C, Lin C, Liu C, et al. (2009) Hepatitis B Virus Core Variants Modify Natural Course of Viral Infection and Hepatocellular Carcinoma Progression. Gastroenterology 137: 1687-1697.

57. Tanaka Y, Mukaide M, Orito E, Yuen MF, Ito K, et al. (2006) Specific mutations in enhancer II/core promoter of hepatitis B virus subgenotypes C1/C2 increase the risk of hepatocellular carcinoma. J Hepatol 45: 646-653.

58. Tangkijvanich P, Sa-Nguanmoo P, Mahachai V, Theamboonlers A, Poovorawan Y (2010) A case-control study on sequence variations in the enhancer II/core promoter/precore and X genes of hepatitis B virus in patients with hepatocellular carcinoma. Hepatology International 4: 577-584.

59. Tong MJ, Blatt LM, Kao JH, Cheng JT, Corey WG (2007) Basal core promoter T1762/A1764 and precore A1896 gene mutations in hepatitis B surface antigen-positive hepatocellular carcinoma: a comparison with chronic carriers. Liver Int 27: 1356-1363.

60. Tatsukawa M, Takaki A, Shiraha H, Koike K, Iwasaki Y, et al. (2011) Hepatitis B virus core promoter mutations G1613A and C1653T are significantly associated with hepatocellular carcinoma in genotype C HBV-infected patients. BMC cancer 11: 458.

61. Truong BX, Seo Y, Yano Y, Ho PT, Phuong TM, et al. (2007) Genotype and variations in core promoter and pre-core regions are related to progression of disease in HBV-infected patients from Northern Vietnam. Int J Mol Med 19: 293-299.

62. Utama A, Purwantomo S, Siburian MD, Dhenni R, Gani RA, et al. (2009) Hepatitis B virus subgenotypes and basal core promoter mutations in Indonesia. World J Gastroenterol 15: 4028-4036.

63. Utama A, Siburian MD, Fanany I, Intan MD, Dhenni R, et al. (2011) Low prevalence of hepatitis B virus pre-s deletion mutation in Indonesia. J Med Virol 83: 1717-1726.

64. Wang HC, Huang W, Lai MD, Su IJ (2006) Hepatitis B virus pre-S mutants, endoplasmic reticulum stress and hepatocarcinogenesis. Cancer Sci 97: 683-688.

65. Wang Y, Liu H, Zhou Q, Li X (2000) Analysis of point mutation in site 1896 of HBV precore and its detection in the tissues and serum of HCC patients. World Journal of Gastroenterology 6: 395-397.

66. Wang Z, Tanaka Y, Huang Y, Kurbanov F, Chen J, et al. (2007) Clinical and virological characteristics of hepatitis B virus subgenotypes Ba, C1, and C2 in China. J Clin Microbiol 45: 1491-1496.

67. Wang X (1996) Study on precore mutation of hepatitis B virus in patients with hepatitis B and hepatocellular carcinoma[Chinese]. CHINESE JOURNAL OF INFECTIOUS DISEASES 01.

68. Wang X (2010) A matched nested case-control study of the relationship between hepatitis B virus pre-S deletion mutations and hepatocellular carcinoma[Chinese]. Journal of Applied Preventive Medicine 01: 5-8.

69. Welschinger R, Kew MC, Viana R, Badri M (2010) T1653 mutation in the enhancer II region of the hepatitis B virus genome in southern African Blacks with hepatocellular carcinoma. Eur J Gastroenterol Hepatol 22: 541-545.

70. Xu L, Qian G, Tang L, Su J, Wang JS (2010) Genetic variations of hepatitis B virus and serum aflatoxin-lysine adduct on high risk of hepatocellular carcinoma in Southern Guangxi, China. Journal of Hepatology 53: 671-676.

71. Yan T, Li K, Su HB, Liu XY, Zang H, et al. (2010) [Analysis the relationship of HBV BCP A1762T/G1764A double mutation with HBV related acute on chronic liver failure]. Zhonghua Shi Yan He Lin Chuang Bing Du Xue Za Zhi 24: 190-192.

72. Yeung P, Wong DK, Lai CL, Fung J, Seto WK, et al. (2011) Association of hepatitis B virus pre-S deletions with the development of hepatocellular carcinoma in chronic hepatitis B. J Infect Dis 203: 646-654.

73. Yin J, Xie J, Liu S, Zhang H, Han L, et al. (2011) Association between the various mutations in viral core promoter region to different stages of hepatitis B, ranging of asymptomatic carrier state to hepatocellular carcinoma. Am J Gastroenterol 106: 81-92.

74. Yin J, Xie J, Zhang H, Shen Q, Han L, et al. (2010) Significant association of different preS mutations with hepatitis B-related cirrhosis or hepatocellular carcinoma. Journal of Gastroenterology 45: 1063-1071.

75. Yuan J, Zhou B, Tanaka Y, Kurbanov F, Orito E, et al. (2007) Hepatitis B virus (HBV) genotypes/subgenotypes in China: mutations in core promoter and precore/core and their clinical implications. J Clin Virol 39: 87-93.

76. Yuan JM, Ambinder A, Fan Y, Gao YT, Yu MC, et al. (2009) Prospective evaluation of hepatitis B 1762(T)/1764(A) mutations on hepatocellular carcinoma development in Shanghai, China. Cancer Epidemiol Biomarkers Prev 18: 590-594.

77. Yuen MF, Tanaka Y, Mizokami M, Yuen JC, Wong DK, et al. (2004) Role of hepatitis B virus genotypes Ba and C, core promoter and precore mutations on hepatocellular carcinoma: a case control study. Carcinogenesis 25: 1593-1598.

78. Yuen MF, Tanaka Y, Shinkai N, Poon RT, But DY, et al. (2008) Risk for hepatocellular carcinoma with respect to hepatitis B virus genotypes B/C, specific mutations of enhancer II/core promoter/precore regions and HBV DNA levels. Gut 57: 98-102.

79. Zhang D, Ma S, Zhang X, Zhao H, Ding H, et al. (2010) Prevalent HBV point mutations and mutation combinations at BCP/preC region and their association with liver disease progression. BMC Infect Dis 10: 271.

80. Zhang F, Shao YF, Gao JD, Xu Y, Liu GT, et al. (2007) [Risk features of HBV in human hepatocarcinogenesis: a nested case-controlled study]. Zhonghua Wai Ke Za Zhi 45: 1482-1484.

81. Zhang KY, Imazeki F, Fukai K, Arai M, Kanda T, et al. (2007) Analysis of the complete hepatitis B virus genome in patients with genotype C chronic hepatitis and hepatocellular carcinoma. Cancer Science 98: 1921-1929.

82. Zhang S (2006) Double-position mutation in the basic core promoter of hepatitis B virus in relation to hepatocirrhosis and liver cancer[Chinese]. Modern Medicine & Health 13: 1924-1925.

83. Zheng JX, Zeng Z, Zheng YY, Yin SJ, Zhang DY, et al. (2011) Role of hepatitis B virus base core and precore/core promoter mutations on hepatocellular carcinoma in untreated older genotype C Chinese patients. J Viral Hepat 18: e423-431.

84. Zhu R, Zhang HP, Yu H, Li H, Ling YQ, et al. (2008) Hepatitis B virus mutations associated with in situ expression of hepatitis B core antigen, viral load and prognosis in chronic hepatitis B patients. Pathol Res Pract 204: 731-742.

85. Zhu Y, Jin Y, Guo X, Bai X, Chen T, et al. (2010) Comparison study on the complete sequence of hepatitis B virus identifies new mutations in core gene associated with hepatocellular carcinoma. Cancer Epidemiol Biomarkers Prev 19: 2623-2630.
