# Supplementary material for: Precore Mutation of Hepatitis B Virus May Contribute to Hepatocellular Carcinoma Risk: Evidence from an Updated Meta-Analysis
Source: PLoS One. 2012 Jun 1;7(6):e38394. doi: 10.1371/journal.pone.0038394 (PMC3365888; doi:10.1371/journal.pone.0038394)
Supplement: Table S2 — Odds ratio for correlation of the other common mutations with HCC risk in the meta-analysis. (DOC) [file pone.0038394.s006.doc]

| **Table S2**. **Summary of the odds ratio for the other common mutations in the meta-analysis** | | | | | | | | |
| --- | --- | --- | --- | --- | --- | --- | --- | --- |
| **Mutations/Subgroup analysis** | **No. of study** | **OR (95%CI)** | **P_OR_^*^** | **I^2^** | **P_H_^＃^** | **Effect model** | **T^§^** | **P_egger_^§^** |
| **A1762T/G1764A (BCP)** | | | | | | | | |
| **Total** | **56** | **3.975 (3.190, 4.954)** | **0.000** | 76.3% | 0.000 | R | -0.84 | 0.406 |
| *Subgroup analysis by ethnicity* | | | | | | | | |
| **Asian** | **50** | **4.112 (3.302, 5.120)** | **0.000** | 73.8% | 0.000 | R |  |  |
| Caucasian | 1 | 1.095 (0.305, 3.927) | 0.889 | / | / | - |  |  |
| African | 3 | 2.772(0.672, 11.443) | 0.159 | 88.7% | 0.000 | R |  |  |
| Mixed | 2 | 4.910(0.496, 48.586) | 0.174 | 88.5% | 0.003 | R |  |  |
| *Subgroup analysis by genotype* | | | | | | | | |
| Genotype A | 3 | 3.667(0.989, 13.602) | 0.052 | 0.0% | 0.458 | F |  |  |
| **Genotype B** | **7** | **4.721 (1.505,14.810)** | **0.008** | **85.4%** | **0.000** | R |  |  |
| **Genotype C** | **13** | **3.744 (2.241, 6.254)** | **0.000** | **67.3%** | **0.000** | R |  |  |
| Genotype D | 2 | 0.947 (0.015, 59.78) | 0.980 | 79.8% | 0.035 | R |  |  |
| *Subgroup analysis by HBeAg* | | | | | | | | |
| **HBeAg negative** | **6** | **4.161 (2.775, 6.239)** | **0.000** | 9.8% | 0.495 | F |  |  |
| **HBeAg positive** | **6** | **6.179(3.268, 11.683)** | **0.000** | 0.0% | 0.246 | F |  |  |
|  | | | | | | | | |
| **X gene** **T1753V** | | | | | | | | |
| Total | **27** | **2.225 (1.689, 2.931)** | **0.000** | 71.8% | 0.000 | R | -0.48 | 0.636 |
| *Subgroup analysis by ethnicity* | | | | | | | | |
| **Asian** | **25** | **2.269 (1.713, 3.005)** | **0.000** | 72.4% | 0.000 | R |  |  |
| African | 1 | 1.222 (0.428, 3.490) | 0.708 | / | / | - |  |  |
| *Subgroup analysis by genotype* | | | | | | | | |
| Genotype B | 2 | 0.667 (0.190, 2.337) | 0.527 | 44.2% | 0.181 | R |  |  |
| **Genotype C** | **8** | **2.229 (1.300, 3.822)** | **0.004** | 82.0% | 0.000 | R |  |  |
| *Subgroup analysis by HBeAg* | | | | | | | | |
| HBeAg negative | 4 | 1.432 (0.416, 4.933) | 0.569 | 77.9% | 0.004 | R |  |  |
| HBeAg positive | 4 | 1.272 (0.572, 2.829) | 0.555 | 0.0% | 0.668 | F |  |  |
|  | | | | | | | | |
| **X gene C1653T** | | | | | | | | |
| Total | **23** | **2.554 (1.946, 3.353)** | **0.000** | 59.5% | 0.000 | R | -0.41 | 0.685 |
| *Subgroup analysis by ethnicity* | | | | | | | | |
| **Asian** | **20** | **2.734 (2.130, 3.509)** | **0.000** | 50.3% | 0.004 | R |  |  |
| African | 1 | 0.480 (0.180, 1.278) | 0.142 | / | / | - |  |  |
| *Subgroup analysis by genotype* | | | | | | | | |
| Genotype A | 2 | 3.143 (0.809,12.209) | 0.098 | 0.0% | 0.568 | F |  |  |
| **Genotype C** | **9** | **2.891 (2.293, 3.646)** | **0.000** | 7.8% | 0.370 | F |  |  |
| Genotype D | 1 | 6.831 (0.801, 58.24) | 0.079 | 0.0% | 0.495 | F |  |  |
| *Subgroup analysis by HBeAg* | | | | | | | | |
| **HBeAg negative** | **4** | **3.341 (1.848, 6.041)** | **0.000** | 25.0% | 0.262 | F |  |  |
| **HBeAg positive** | **4** | **2.345(1.123, 4.898)** | **0.023** | **4.4%** | **0.371** | F |  |  |
| ***Subgroup analysis by disease group*** | | | | | | | | |
| **A1762T/G1764A** | **27** | **5.455(3.882, 7.666)** | **0.000** | 76.8% | 0.000 | R | -1.67 | 0.10 |
| **G1896A** | **22** | **1.851 (1.267, 2.704)** | **0.001** | 81.5% | 0.000 | R | -1.54 | 0.139 |
| **T1753V** | **11** | **2.774 (1.921, 4.006)** | **0.000** | 38.5% | 0.092 | F | -0.93 | 0.378 |
| **C1653T** | **8** | **2.586 (1.530, 4.371)** | **0.000** | 67.1% | 0.003 | R | 0.35 | 0.741 |

* P_OR_ value for the odds ratio

# P_H_ the P value of the Heterogeneity test

§ T for Egger’s test, P_egger_, the P value for Egger’s test

※ R for the Random-effects model, F for the Fixed-effects model.

♂ Adjustment for Heterogeneity by omitting the most obvious outliers.
